# Biomedical Evaluation of *Lansium parasiticum* Extract-Protected Silver Nanoparticles Against *Haemonchus contortus*, a Parasitic Worm

**DOI:** 10.3389/fmolb.2020.595646

**Published:** 2020-12-17

**Authors:** Vanshita Goel, Pawandeep Kaur, Lachhman Das Singla, Diptiman Choudhury

**Affiliations:** ^1^School of Chemistry and Biochemistry, Thapar Institute of Engineering and Technology, Patiala, India; ^2^Department of Veterinary Parasitology, Guru Angad Dev Veterinary and Animal Sciences University, Ludhiana, India; ^3^Thapar Institute of Engineering and Technology-Virginia Tech (USA) Center for Excellence in Material Sciences, Thapar Institute of Engineering and Technology, Patiala, India

**Keywords:** phytochemical analysis, antioxidant activity, reactive oxygen species, nitric oxide synthase, silver nanoparticles, *Haemonchus contortus*, *Lansium parasiticum*

## Abstract

Here we show the novel anti-helminthic potential of *Lansium parasiticum* aqueous extract-protected silver nanoparticles (LAgNPs) against albendazole-resistant gastrointestinal parasite *Haemonchus contortus*. LAgNPs showed LD_50_ values of 65.6 ± 32.8 nM (12 h), 139.6 ± 39.9 nM (12 h), and 64.3 ± 8.5 nM (24 h) against adult male, female, and L3 larvae, respectively. LAgNPs was also quite effective in inhibiting egg hatching, with an IC_50_ value of 144.4 ± 3.1 nM at 48 h of exposure. Exposure to LAgNPs generated oxidative stress and mediated physical damage in the worms' tissue. A sharp increase in reactive oxygen species and nitric oxide synthase levels was prominent due to LAgNPs' exposure. In response to oxidative stress, a sharp increase of stress-responsive enzymes' activity, like catalase, superoxide dismutase, and glutathione peroxidase activity, along with the concentration of glutathione, was observed in worm tissue, which indicated a LAgNP-responsive alteration of metabolism. The results give rise to the opportunity for the development of alternative treatment for drug-resistant parasitic worms.

## Introduction

Parasitic nematode infection in the gastrointestinal tract is the world's most common economically important infectious disease in humans and animals (Bethony et al., 2006; Knopp et al., 2012). Almost 24% of the total human population and almost 90–100% of the animal population are infected with gastrointestinal parasitic infections (Harhay et al., 2010; Utzinger et al., 2012; Besier et al., 2016). The infections are prevalent in tropical environments like sub-Saharan Africa, North America, and South and East Asia. Infants and school-age children and pregnant women are the most vulnerable group, where helminth infection may cause life threats (World Health Organization, 2002).

*Haemonchus contortus*, a highly infectious gastrointestinal parasitic nematode responsible for acute anemia, hemorrhagic gastroenteritis, diarrhea, depression, *etc*., in mainly, but not limited to, ruminants leads to reduction of livestock production and milk and meat production, which cause a huge economic loss worth 120 billion USD worldwide every year (Horton, 2003; Kaplan, 2004; Zajac, 2006; Tariq, 2015; Singh et al., 2017). *H contortus* is transmitted through infected soils in various species, causing infection including in humans (Vercruysse et al., 2011; Sinnathamby et al., 2018). *H. contortus* infection in the blood has also been reported in many animals, including goats, sheep, rabbits, humans, *etc*., causing inflammation and immune-modulations (Tak et al., 2012; Rehman et al., 2015; Wang et al., 2017, 2019). At present, there are only a handful of drugs available (like benzimidazole, imidazothiazole, and ivermectin) for helminth treatment, and the development of chemoresistance is a major challenge of this field (Easwaran et al., 2009; Mullner et al., 2011; Kaplan and Vidyashankar, 2012; Das et al., 2015; Dixit et al., 2017; Furgasa et al., 2018). In 1964, the first report of chemo-resistance in *H. contortus* came against benzimidazole drug (Cawthorne and Whitehead, 1983). After that, a few more reports were published on the resistance against different anti-helminthic drugs. In 1983, Cawthorne and Whitehead reported on the resistance for the inhibition of microtubule polymerization by benzimidazole in *H. contortus* (Cawthorne and Whitehead, 1983). Moreover, in 1979, Sangster et al. reported the development of resistance against imidazothiazoles (Sangster et al., 1979). Gill (1996) first ever reported the resistance of parasites to albendazole. Furthermore, Hoti et al. (2009) and Ram et al. (2007) reported the resistance in presently available drugs (albendazole, levamisole, fenbendazole, etc.). The prevalence of infection, shortage of drugs, and increasing resistance against existing drugs bring us a situation of the urgent need for new drugs.

The use of natural resources to treat various communicable and non-communicable diseases is a practice from pre-historical ages (Chintamunnee and Mahomoodally, 2012). Various herbal formulations derived from chamomile, nettle, Shatavari, garcinia, neem, karela, pippali, ashwagandha, ajwain, fenugreek, wormwood, etc., are documented in Indian, Chinese, Vietnamese, and African traditional medicine (Croizier, 1968; Borins, 1987; Sofowora, 1996). These herbal formulations are rich in various secondary metabolites, including various polyphenolic compounds like alkaloids, flavonoids, tannins, etc., which are documented to have anti-helminthic activity (Mushtaq et al., 2018). Despite the prevalence of helminthic infections, scarcity of medication, and profound documentation of traditional medicines, only a handful of those natural resources have ever been explored for their potential in modern medicine. Eguale et al. (2011) performed *in vitro* anthelmintic activity using hydro-alcoholic and aqueous extracts of *Coriandrum sativum* seeds for the investigation of inhibition of egg hatch and growth in the adult stage. Maciel et al. (2006) used the ethanol extract of the leaf and seed of Melia azedarach plant on *H. contortus* larval growth inhibition and egg hatching to confirm its antihelmintic activity. Pessoa et al. (2002) used *H. contortus* eggs for checking Ocimum gratissimum anti-helminthic activity on the development of larva from eggs, whereas Tomar and Preet (2017) synthesized silver nanoparticles using *Azadirachta indica*, an aqueous extract, to analyze the anti-helminthic activity against *H. contortus*. Goel et al. (2020) used an aldehyde derivative of cumin and performed *in vitro* anti-helminthic activity to examine the inhibition of egg hatch and growth in the larval and adult stage, along with the generation of reactive oxygen species and enzymatic activities, to confirm the mechanism of action. Kumarasingha et al. (2016) performed anti-helminthic activity on *H. contortus* using various ethnomedicinal plants like *Lansium domesticum, Linariantha bicolor*, and *Tetracera akara*. Ahmed et al. (2020) used traditional medicinal plants like *Artemisia herba-aba* and *Punica grantum* to study the anti-helminthic activity of *H. contortus* worms. Moreover, Zenebe et al. (2017) studied the *in vitro* anti-helminthic activity of gastrointestinal worms with the crude extracts of *Cissus quadrangularis* and *Schinus molle*.

Furthermore, the recent advancement of nanotechnology in the field of healthcare came up with new hope for the management of infectious diseases like helminth infections. Rashid et al. (2016) performed anti-helminthic activity using *Momordica charantia*-coated silver nanoparticles against gastrointestinal worms. Shakir et al. (2015) used cadmium-coated nanoparticles and observed the antiworm for parasitic worms. Kar et al. (2014) used *Nigrospora oryzae*-coated gold nanoparticles to check the anti-helminthic activity of cestodes. Barbosa et al. (2019) showed the nematicidal activity of silver nanoparticles coated with *Duddingtonia flagrans* on the larval stage of *Ancylostoma caninum*. André et al. (2020) performed anti-helminthic activity on the adult stage of *H. contortus* using carvacrol-coated chitosan nanoparticles. Ejaz et al. (2017) used *Artemisia vulgaris* reduced silver nanoparticles to check the anti-worm effect on various nematodes. Rehman et al. (2019) used silver nanoparticles coated with *Tribulus terrestris* to observe the anti-helminthic activity of flukes. Preet and Tomar (2017) bio-fabricated silver nanoparticles using *Ziziphus jujube* for *in vitro* adulticidal and egg hatch assay for the same worm.

*Lansium parasiticum* is a widely grown plant in the northeastern Himalayan regions of India, Bangladesh, Myanmar, etc., used mainly for eating and timber purposes (Morton, 1987; Hassler, 2019). The fruits of the plant grow during July and August season and are rich in polyphenolic compounds like minerals (potassium, sodium, manganese, phosphorus, etc.), organic acids (maleic acid, citric acid, and glycolic acid), and vitamins (vitamin C, vitamin A, thiamine, riboflavin, niacin, etc.) (Venkatachalam, 2020). The seed extract of *L. parasiticum* is known for its use in deworming, ulcer medication, dysentery, and malaria in traditional medicine (Tanaka et al., 2002; Abdullah et al., 2005; Sunpapao et al., 2016; Ruhisha and Choudhury, 2017; Potipiranun et al., 2018; Ramadhan et al., 2018). Here we show the novel anti-helminthic activity of the fruit extract-protected silver nanoparticles (LAgNPs) for the first time, which opens the scope of exploring this plant for the development of modern medicine.

## Materials and Methods

### Materials

AgNO_3_ was procured from Sigma Aldrich, USA. RPMI, Dulbecco's Modified Eagle Medium (DMEM), antibiotics, and fetal bovine serum were purchased from Himedia Ltd., India. All the other reagents of analytical grade were purchased from Loba Chemie, India.

### Collection of Fruits of *L. parasiticum*

Fresh fruits of “Latka” were collected during June–July from the local market in the foothills of eastern Himalaya. The fruits were washed several times in water, and then the cover of the fruits, pulps, and seeds were separated from each other and oven-dried for 8–10 days at 60°C. Dried tissues were ground into powder and stored at room temperature in air containers for further use.

### Preparation of *L. parasiticum*-Protected Silver Nanoparticles

An aqueous extract of *L. parasiticum* (ALE, pH 7.0) was prepared by boiling 3 g of the powder of dried pulp in 100 ml distilled water for 1 h. After cooling, the extract was filtered using Whatman no. 1 filter paper and stored at 4°C for future experiments.

AgNP synthesis was carried out by using ALE as a reducing and capping agent. For the preparation of nanoparticles, 240 μM AgNO_3_ was added dropwise in 5,000 μl of ALE (pH 7.0) and was kept under the sunlight (bright) in slow stirring condition (150 rpm) for 30 min. In the presence of sunlight, the reduction of Ag^+^ ions into Ag^0^ took place, resulting in a change in color from colorless to pale yellow and finally reddish-brown. Thus, the synthesized aqueous *L. parasiticum*-protected AgNPs were designated as LAgNPs. The particles were washed thrice using a 1× volume of sterile 0.05% NaCl solution (2,000 rpm) and then suspended in 1× volume of 0.05% NaCl solution for characterization and further use (Heyne, 1987; Ahmed et al., 2016).

### Physical Characterization of LAgNPs

Thus, the formed LAgNPs were characterized using surface plasmon resonance (SPR) pattern under UV–vis spectra ranging from 300 to 700 nm at different time points (0–30 min) using a Shimazu UV-2600 spectrophotometer. The average hydrodynamic diameter and stability of biogenic LAgNPs were determined using dynamic light scattering (DLS) and zeta potential studies by Microtrac's dynamic light scattering model Nanotrac. Shape, size, and elemental composition (energy-dispersive X-ray spectroscopy; EDS) were examined by transmission electron microscopy (TEM) using JEOL-2100, JEM USA. For TEM–EDS analysis, particles were coated on a carbon-coated 400 mesh copper grid. To analyze the involvement of various functional groups working as a surface-protecting agent on LAgNPs, Fourier-transform infrared spectroscopy (FTIR) analysis was carried out using Agilent Resolution Pro-carry 660 machine.

### Preparation and Physical Characterization of Citrate-Coated AgNPs

Five milliliters of 0.01 M AgNO_3_ was heated to boiling. To this solution, 0.5 ml of 1% trisodium citrate (pH 5.0) was added drop by drop. During the process, the solution was mixed vigorously and heated until the change in color was evident (brownish-yellow). Then, it was removed from the heating device and stirred until cooled to room temperature. After synthesis, the particles were washed with distilled water twice and suspended in 1× phosphate-buffered saline (PBS, pH 7.0) for storage and further experiments.

The citrate-coated AgNPs were characterized using SPR pattern under UV–vis spectra ranging from 300 to 700 nm using a Shimazu UV-2600 spectrophotometer. The average hydrodynamic diameter and stability of biogenic LAgNPs were determined using DLS and zeta potential studies by Microtrac's dynamic light scattering model Nanotrac.

### Toxicity Profiling of LAgNPs on Normal Human Cells

Human embryonic kidney cancer cells HEK239 were maintained and test-cultured in DMEM, supplemented with 10% fetal bovine serum, in the presence of 5% CO_2_ at 37°C. The cells were seeded in 96-well plates and treated with LAgNPs at ~70% confluence for 24 h. After the treatment, 2 μM 3-(4, 5-dimethylthiazol-2-yl)-2, 5-diphenyl tetrazolium bromide (MTT) solution was added, and the mixture was incubated for 3 h at 37°C. The final results were then observed by dissolving formazan crystals in dimethyl sulfoxide (DMSO) and measuring the absorption at 570 nm (Choudhury et al., 2013; Datta et al., 2017).

### Collection and Identification of *H. contortus* From Ruminants

Adult *H. contortus* worms were isolated from the abomasa of infected ruminants collected from the slaughterhouses of Ludhiana, Punjab, India. The abomasa were thoroughly washed with running water to remove the excreta. The internal content of the abomasum was collected and washed several times using a sieve. The *H. contortus* adults were picked and identified using a fine brush and were readily transported in 1× PBS at pH 7.4 (Goel et al., 2020).

### Isolation of Eggs From Adult Females

The females were identified based on their morphology, washed, and incubated with 1× PBS. Furthermore, a freshly prepared saturated saline solution was added, and centrifugation was done at 11,000 × *g* for 15 min. The topmost layer of fluid containing eggs was procured. Thereafter, a thick suspension was prepared and then finally diluted using 1× PBS saline to get a concentration of 200 eggs/ml using the McMaster technique and stored at 4°C for further use (Goel et al., 2020).

### Collection and Harvesting of Infective Larval Stage (L3)

The homogenized mixture-infected fecal samples were incubated for 7 days in the dark at room temperature (25°C). Furthermore, for better oxygenation, regular supplementation of water was done, which kept the mixed culture wet. Thereafter, the homogenized mixture was analyzed, and saline containing infective larvae was collected from the mixed fecal culture and stored at 4°C for future tests. The larvae were disinfected by the addition of 0.2% sodium hypochlorite for the removal of adherent bacteria (Goel et al., 2020).

### Adult Motility/Morbidity Test

Adult motility/morbidity test for *H. contortus* was performed using a set of 15 male and female worms each in the presence of LAgNPs at different concentrations (15.8, 31.7, 63.5, and 158.7 nM) along with untreated, albendazole- (9.4 μM), and citrate-coated AgNPs (140 nM) and Ag^+^ (100 μM) in 1× RPMI medium and incubated for 24 h at 37°C. After the incubation, the mortality of the worm was confirmed in response to physical stimuli. The observations for paralysis and death time were recorded at regular time intervals (0, 1, 3, 6, 12, and 24 h). Furthermore, the morphological changes after the treatment were analyzed by microscopic examination at 40× magnification. The morbidity of adult worms was analyzed after visualizing their movement (for 60 s) after moderate agitations. Thereafter, the mortality of the treated worms was confirmed in response to heat shock treatment in 0.8% saline at 50°C for 10 s, and these were consequently stained with Lugol's stain to observe the morphological changes (Goel et al., 2020).

### Larval (L3) Viability Assay

The larval (L3) viability test of *H. contortus* was executed by picking ~25–30 larvae in 200 μl of RPMI medium in 96-well plates in the presence of LAgNPs at different concentrations (15.8, 31.7, 63.5, and 158.7 nM) along with untreated, Ag^+^ (100 μM) and Alb (9.4 μM), and these were kept for incubation for 24 h at 27°C. Thereafter, direct microscopic examinations were done after the incubation for mobility (for 20 s), morphology, and reaction to physical stimuli to analyze the viability of the worms. Moving and coiled larvae were observed as live; otherwise, spread larvae were considered as dead. Subsequently, Lugol's staining technique was performed to examine the changes in morphology after the treatment. The viability percentage of larvae L3 (% ML3) was calculated by using the following formula:

(1) $$\begin{array}{l} \%\text{ ML}3=[(\text{number L}3\text{ dead}/(\text{number of L}3\text{ dead}\\ +\text{ number of live L}3)]*100 \end{array}$$

### Egg Hatch Assay

The egg hatch assay was performed by taking 1 ml of egg suspension, which contained ~200 eggs/ml, into a 24-well microtiter plate. The experiment sets were treated as detailed above. Afterward, the microtiter plate was incubated at 28°C for 48 h. After 48 h of treatment, Lugol's iodine drop (10 μl) was added in each well for the disruption of the inhibition process, and then the final count of the total number of eggs hatched or inhibited was obtained directly under the microscope. After that, the final count of eggs inhibited from hatching or the larvae present were counted and calculated to determine the total percentage of inhibition (survival) of eggs hatched (%EHI) by using the following formula:

(2) $$\begin{array}{l} \%\text{ EHI}=[\left(\text{number of larvae}/\text{eggs hatched}\right)\\ /(\text{total number of eggs})]*100 \end{array}$$

### MTT Assay to Determine the Cell Viability of *H. contortus* and Human

Both the worm tissue (100 mg) and human normal kidney cells (HEK293) were incubated with various concentrations of LAgNP (15.8 to 158.7 nM) along with untreated and Alb-treated (9.4 μM) and Ag^+^-treated (100 μM) controls. For *H. contortus*, the cells were treated for 3 h, and the HEK293 cells were treated for 24 h. After the treatment, 2 μM MTT solution was added to each well, and the samples were further incubated for 3 h. Absorption data were collected at 595 nM after dissolving the formazan crystals in DMSO (Kaur et al., 2019; Mehta et al., 2019).

### SEM Studies for Monitoring Physical Damage of Warm

To monitor the physical damage on adult *H. contortus* after the treatment with LAgNPs, scanning electron microscopy was used. The infectious helminths were treated with (158.7 nM) of LAgNPs and a set of untreated controls, Alb (9.4 μM) and Ag^+^ (100 μM) for 12 h, and then washed with 1× PBS for further coatings. Before the gold coatings, dehydration of treated and control worms was done using 50 to 100% gradient concentrations of ethanol. Thereafter, the dehydrated worms were coated by applying gold (15 μm) under a specific vacuum condition. After the gold coating, the samples were observed using an SEM (JEOL JSM 6490LV) at an electron accelerating voltage of 15 kV (Puspitasari et al., 2016).

### Measurement of Total Protein Concentration

By using the Bradford method, total protein concentration was measured in treated and control *H. contortus* worms. For the Bradford assay, 250 mg of control and treated worm tissue was homogenized using 500 μl of radioimmunoprecipitation assay buffer. A total protein concentration was estimated after centrifugation at 10,000 rpm for 15 min against the bovine serum albumin (BSA) standard curve (Hammond and Kruger, 1988).

### Measurement of Stress Generation Due to Reactive Oxygen Species

The stress caused by LAgNPs conducted a cellular response, and a balance between the production of antioxidant defenses and ROS (free radicals) was observed. The nematode *H. contortus* was treated with different concentrations of AgNPs (15.8, 31.7, 63.5, and 158.7 nM) for 3 h, and thereafter analysis of the treated worm was done. Particular parts of the worms were then identified, sectioned, and washed with distilled water. Furthermore, the sectioned parts were treated with a fluorogenic dye, 100 nM of 2′,7′-dichlorofluorescein diacetate (DCFDA), and incubated in the dark for 20 min at 37°C. The incubated samples were washed using distilled water again to remove the excess of DCFDA, and the slide was fixed for fluorescent imaging by using an inverted Dewinter fluorescence microscope. The posterior and the anterior ends and the complete body structure (differentiating the male and the female) were mainly analyzed for the comparison of ROS generation of the treated helminths against a set of controls, Alb, and Ag^+^ (Datta et al., 2019).

### Determination of Nitric Oxide Synthase

The interactions between the oxygenase domains and reductase have several layers that regulate the involvement of enzymatic activities, expressed by NOS. Therefore, loss of enzymatic activities causes the hindrance of superoxide dismutase (SOD) and reduction of glutathione and allows the increase in ROS, which, in turn, would further improve the level of toxicity of NOS. Adult *H. contortus* worms were treated with LAgNPs for 3 h, and the total protein concentration was determined. Thus, for the generation of nitric oxide synthase, the method of Höglund et al. (2015) for NOS was done by adding 0.1 ml of Greiss reagent. Then, the test reaction was observed spectrophotometrically at 540 nm at different times, i.e., 30 min and 6, 12, and 24 h (Ulker et al., 2010).

### Measurement of Antioxidant Enzyme Activity

The free radicals generated during normal metabolic functions start reacting with the cellular molecules, which further induce the defense oxidative damage mechanisms. The following mechanisms include the damage of the thiol-specific antioxidants, CAT, GPx, and SOD.

### Estimation of Superoxide Dismutase

After total protein extraction, 50 mg (volume, 1 ml) of protein was incubated with 1.4 ml of reaction mixture aliquot [phosphate buffer, 50 mM; pH 7.4; methionine, 20 mM; hydroxylamine hydrochloride, 10 mM; 1% (v/v) Triton X-100, ethylenediaminetetraacetic acid (EDTA; 50 mM)] for 5 min at 37°C. After this, 50 μM riboflavin was added and exposed for 10 min to a 200-W fluorescent lamp. Thereafter, 1 ml Greiss reagent was added, and absorbance was taken at 543 nm (Kong et al., 2012; Hadwan and Abed, 2016). The SOD activity was determined using the following equation:

(3) $$\begin{array}{l} \text{Enzymatic activity}=\frac{\text{Volume of the assay}\times \text{absorbance}\times \text{dilution factor}}{\text{Time}\left(\text{in minutes}\right)\times \text{volume of the enzyme}} \end{array}$$

### Estimation of Catalase

*H. contortus* treatment and protein extraction were done as mentioned in the discussion above. Thereafter, 0.2 M H_2_O_2_ reaction mixture was prepared at pH 7; phosphate buffer was added to the pre-incubated 1 ml (50 mg/ml) protein of worm along with the control. Then, dichromate acetic acid solution (v/v) was added after 0, 30, 60, and 90 s; after that, heating of the above-mentioned mixture was carried out for 10 min, and changes in the color of the solutions were observed at 610 nm absorbance. Negative control was made using 100 mg/ml BSA solution (Hadwan and Abed, 2016). Total enzyme activity was calculated using Equation (3).

### Glutathione Peroxidase Assay

The glutathione peroxidase reduction ability was calculated using 50 mg extracted protein of the treated helminths. Then, 0.1 ml sodium azide (NaN_3_), 0.2 ml EDTA, and 0.2 ml Tris buffer were added in the 0.5 ml homogenate of worm tissue. After this, 0.2 ml glutathione and 0.1 ml H_2_O_2_ were incubated for 10 min at 37°C. Then, 0.5 ml trichloroacetate was added, and the mixture was centrifuged to eliminate the pellet. Then, the supernatant was collected, and DTNB and Na_2_HPO_4_ were added. Absorbance at 412 nm was recorded (Antunes and Cadenas, 2000).

### Glutathione Reductase Assay

The relation between oxidative stress and the increasing concentration of LAgNPs is direct. The glutathione reduction was calculated using tissue homogenate (1.0 ml of 10%) and was then precipitated out using 4.0 ml metaphosphoric acid. After centrifugation, the precipitate was removed, and then 1 ml of DTNB, 2 ml Na_2_HPO_4_, and 2 ml of supernatant were added. Absorbance was observed at 405 nm against the blank (Ulker et al., 2010; Höglund et al., 2015).

### Statistical Analysis

The complete data were presented as the mean of at least three independent experiments. Statistical analysis of data was conducted by Student's *t*-test. Two measurements were considered as statistically significant if the corresponding *p* < 0.01.

## Results and Discussion

### Evaluation of LAE-Protected AgNPs' Physical Properties

**Figure 1:**
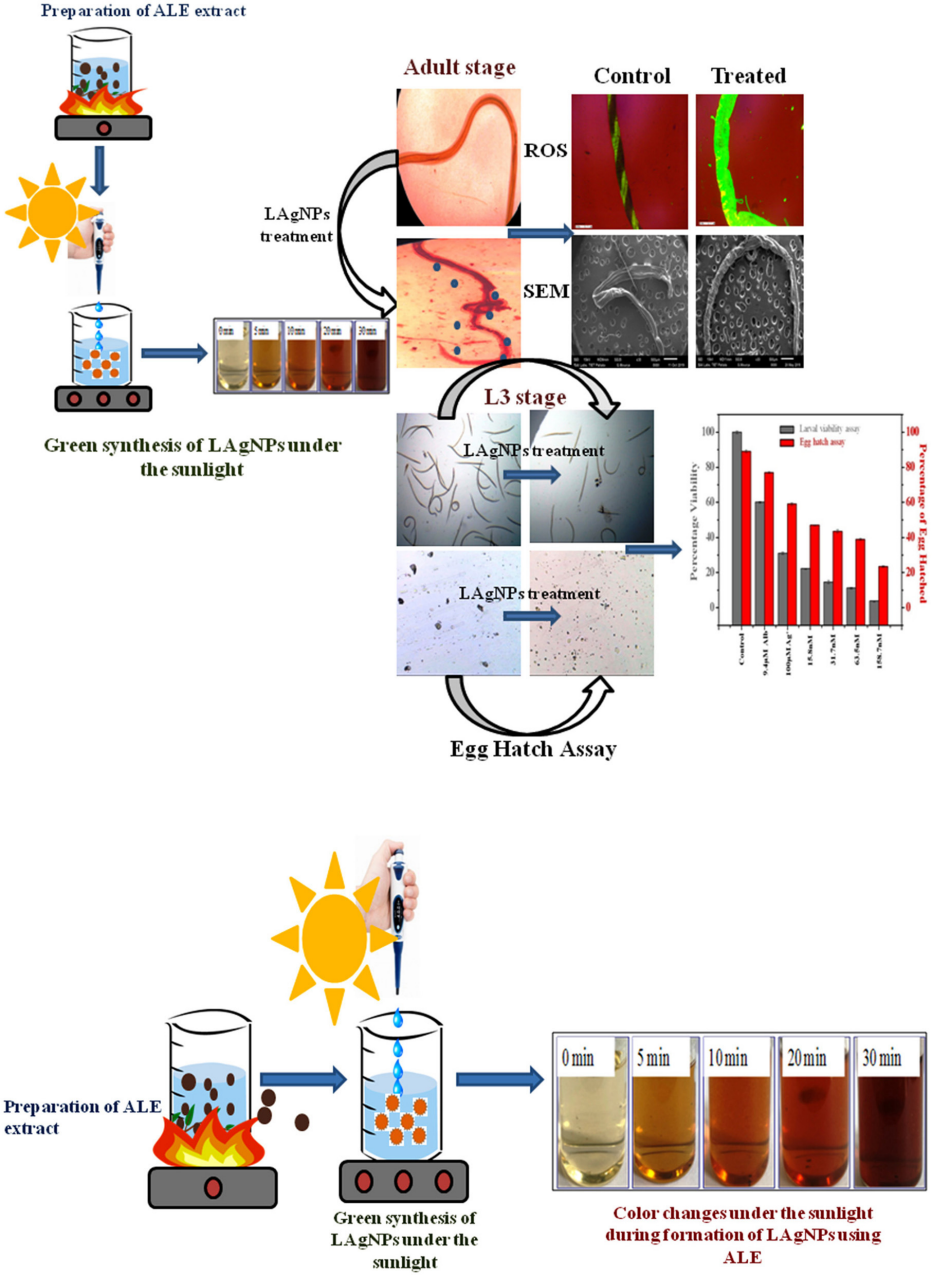
Schematic representation of the preparation of aqueous *Lansium parasiticum* pulp extract (ALE) and the synthesis of *L. parasiticum*-protected silver nanoparticles (LAgNPs) under the sunlight. Visible color changes under the sunlight during the formation of LAgNPs at different time intervals, 0–30 min.

**Figure 2:**
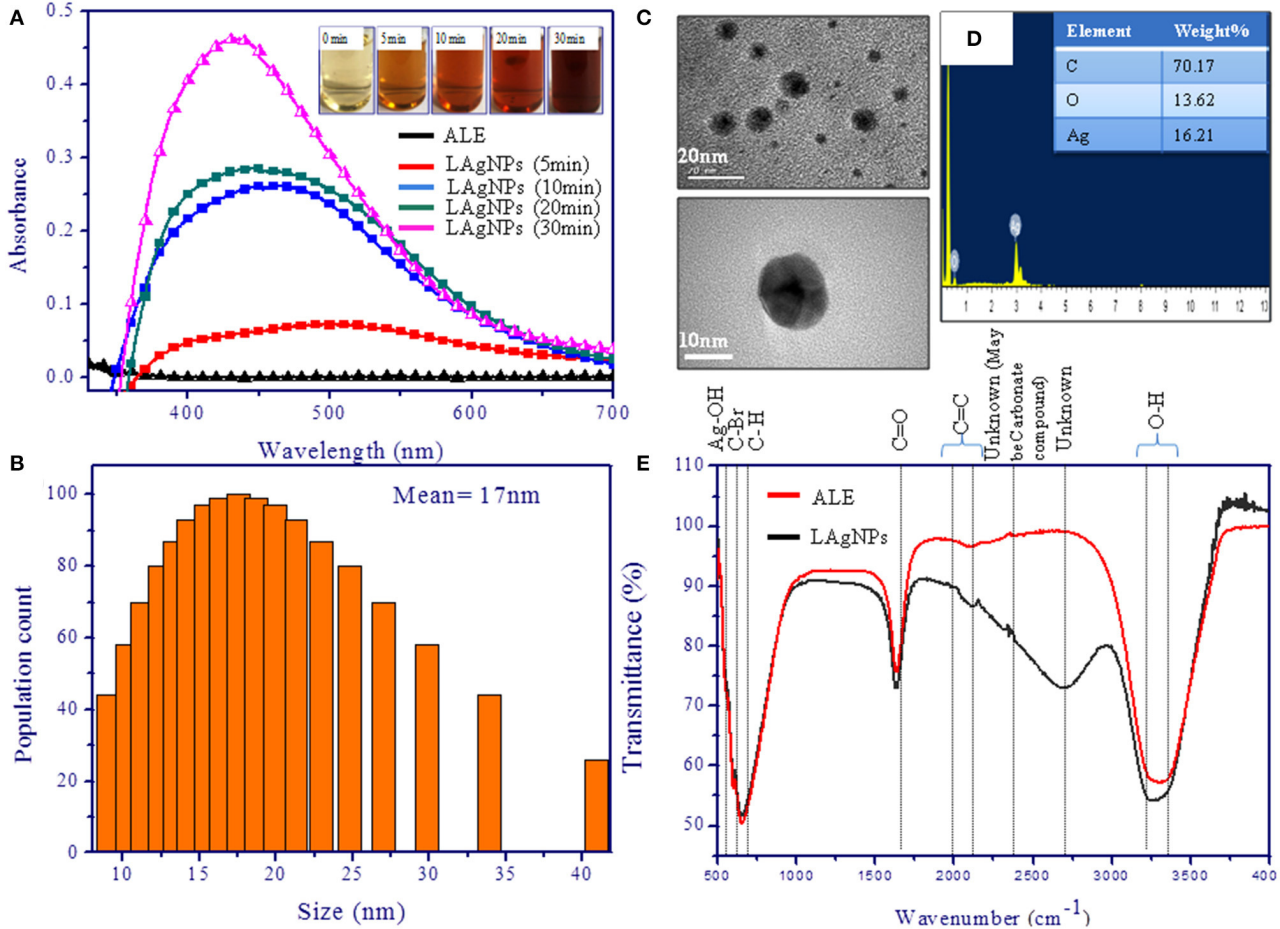
Characterization of silver nanoparticles (AgNPs). **(A)** Surface plasmon resonance spectra of photo-reduced AgNPs at different time intervals. The spectra were scanned in the range of 300–700 nm. **(B)** Dynamic light scattering study showing the distribution of hydrodynamic diameter and disparity of the *Lansium parasiticum*-protected silver nanoparticles (LAgNPs). The hydrodynamic size obtained was 17 nm. **(C)** Transmission electron microscopic studies for the size and the shape of the AgNPs. The inset is showing the mean values of the LAgNPs. **(D)** Energy-dispersive X-ray spectroscopy studies showed the elemental composition of the LAgNPs. **(E)** Fourier-transform infrared spectroscopy studies confirmed the changes in functional groups, including plant extract and LAgNPs.

The formation of LAgNPs under the sunlight was visibly observed by color change (Figure 1) and thereafter was confirmed by observing by SPR. Upon the formation of LAgNPs, a time-dependent blue shift in the SPR peak position (absorption maximum) was observed from 450 nm. A saturation of peak intensity and the completion of the SPR shift at 435 nm were observed after 30 min of incubation (Figure 2A).

DLS data showed the formation of monodispersed nanoparticles with an average hydrodynamic diameter of 17 ± 9 nm (Figure 2B). Furthermore, the zeta potential analysis of LAgNPs showed the formation of stable particle surface charge of −21 mV.

TEM studies confirmed the synthesis of spherical nanoparticles with an average diameter of ~16 ± 5 nm (Figure 2C). Images of LAgNPs at higher magnifications are presented in the inset. Elemental analysis using EDS showed the involvement of 16.21% silver by weight in the LAgNPs. As a typical absorption peak of metal silver, synthesis showed an optical absorption peak at 3 keV (shown in Figure 2D).

In addition to these studies, to check the involvement of different functional groups of ALE in the synthesis of LAgNPs, FTIR studies were performed. The surface protection of AgNO_3_ was observed with the formation of Ag–OH (557 cm^−1^) bond in LAgNPs. Along with this, the other bonds C=C and O–H shift from 2,118.5 and 3,299 cm^−1^ in ALE to 2,155 and 3,245 cm^−1^, respectively, in LAgNPs, indicating that these functional groups may play an important role in the reduction and capping of LAgNPs (Figure 2E). The band positions and respective shifts in FTIR along with the references are given in Table 1 (Alkilany et al., 2015; Gayathri et al., 2015; Jyoti et al., 2016; Paulkumar et al., 2017; Senthil et al., 2017).

**Table 1:** Fourier-transform infrared spectroscopy analysis for functional groups for aqueous *Lansium parasiticum* pulp extract (ALE) and silver nanoparticles coated with *Lansium parasiticum*.

**Functional groups**	**Wavenumber (cm**^****−1****^**)**		**References**
	**ALE**	**LAgNPs**	
Ag–OH	–	557	Gayathri et al., 2015
C–Br stretching	609.8	609.8	Jyoti et al., 2016
C–H stretching	663.3	648.4	Jyoti et al., 2016
C=O stretching	1,637	1,637	Paulkumar et al., 2017
C=C stretch	2,118.5	2,155	Senthil et al., 2017
Unknown (maybe carbonate compound)	2,354	2,348.4	
Unknown	–	2,695	
O–H stretch	3,299	3,245	Alkilany et al., 2015

### Evaluation of Citrate-Coated AgNPs' Physical Properties

The formation of citrate-coated AgNPs under boiling conditions was observed by a color change and thereafter confirmed by observing SPR, with an extinction maximum at 425 nm. DLS data showed the formation of monodispersed stable citrate-coated AgNPs with an average hydrodynamic diameter of 23.5 ± 2.1 nm and with a surface charge of −19 mV (Zeta potential analysis). The figures are discussed in the supplementary section with Supplementary Figure 2.

### Toxicity of LAgNPs on Human Normal Kidney Cells

LAgNPs are shown to cause mild to moderate loss of human normal kidney cell viability where it showed significant toxicity of *H. contortus* cells. The detailed results are discussed in the supplementary section with figures and tables (Supplementary Table 3, Supplementary Figures 3A,B).

### Assessment of Anti-helmintic Activities

#### Adult Motility and Morbidity Assay

The effect of LAgNPs on the viability of *H. contortus* is concentration dependent. Approximately 60 and 20% of male and female worms, respectively, got paralyzed in 1 h; 100% of male worms and 80% of female worms died within 12 h of treatment with 158.7 nM LAgNPs. The details of the death and time of paralysis are given in Table 2. Whereas, in citrate-coated AgNPs 0% of male and female worms got paralyzed in 1 h, only 26% of male worms and 11.3% of female worms died after 12 h of citrate AgNP treatment. Thus, LAgNPs were more effective and showed higher toxicity against parasitic worms, whereas citrate particles showed comparatively lesser toxicity. Therefore, we may infer that silver nanoparticles are toxic against parasitic worms, and the effect further got enhanced due to the active principal component(s) present in the *L. parasiticum* extract. The LD_50_ values were found to be 65.6 ± 32.8 and 139.6 ± 39.9 nM for adult male and female worms, respectively, for 12 h of treatment (Supplementary Figures 4A,B for male and female adult worms, respectively).

**Table 2:** Paralysis and death time analysis of AgNPs on adult *Haemonchus contortus*.

**Time of exposure**	**Paralysis time**								**Death time**							
	**Control (RPMI)**	**Alb**	**Ag^**+**^**	**Citrate AgNPs**	**15.8**	**31.7**	**63.5**	**158.7**	**Control (RPMI)**	**Alb**	**Ag^**+**^**	**Citrate AgNPs**	**15.8**	**31.7**	**63.5**	**158.7**
**Male worms**																
0 h	–	–	–	–	–	–	–	–	–	–		–	–	–	–	–
0.5 h	–	–	–	–	–	–	–	3	–	–	–	–	–	–	–	–
1 h	–	–	–	–	–	–	6	6	–	–	–	–	–	–	–	–
3 h	–	–	–	1	–	–	9	12 ± 0.5	–	–	–	–	–	–	–	–
6 h	–	3 ± 0.5	–	4 ± 0.5	–	3	12 ± 0.5	9	–	–	–	–	–	–	–	6
12 h	–	6 ± 0.5	3	15 ± 0.5	6 ± 0.5	6	9	0	–		–	4 ± 1	3	7 ± 0.7	6	15
24 h	1	0	6	15 ± 0.5	6 ± 0.5	3 ± 0.5	0	0	–	15	9	15 ± 0	15	12 ± 0.5	15	15
**Female worms**																
0 h	–	–	–	–	–	–	–	–	–	–	–	–	–	–	–	–
0.5 h	–	–	–	–	–	–	–	–	–	–	–	–	–	–	–	–
1 h	–	–	–	–	–	–	–	3 ± 0.5	–	–	–	–	–	–	–	–
3 h	–	–	–	–	–	–	–	9 ± 0.5	–	–	–	–	–	–	–	–
6 h	–	–	–	–	–	–	6	15	–	–	–	–	–	–	–	–
12 h	–	–	–	5.7 ± 0.5	3	3	6	6	–	3	–	1.7 ± 1	–	3	9	9
24 h	1	–	3	10.7 ± 1.3	3	6	0	0	–	6	–	5.5 ± 1.3	3	9 ± 0.5	15	15

*Adult H. contortus worms were treated with LAgNPs at different dose concentrations (15.8, 31.7, 63.5, and 158.7 nM), Alb (9.4 μM), citrate-coated AgNPs (140 nm), and Ag^+^ (100 μM) in RPMI media for 24 h. Paralysis and death of the worms were analyzed at various time points in between. After the treatment, the worms were first noted for their movement. Therefore, if no movement was observed, then they were exposed to warm saline (0.8%) solution (50°C) for 1 min to stimulate movement. The counts were taken for the number based on the worm's movement. If the previously immobile worm starts moving upon exposure to warm saline, then it was considered as paralyzed; otherwise, it was considered dead*.

#### Larval Morbidity Assay

**Figure 3:**
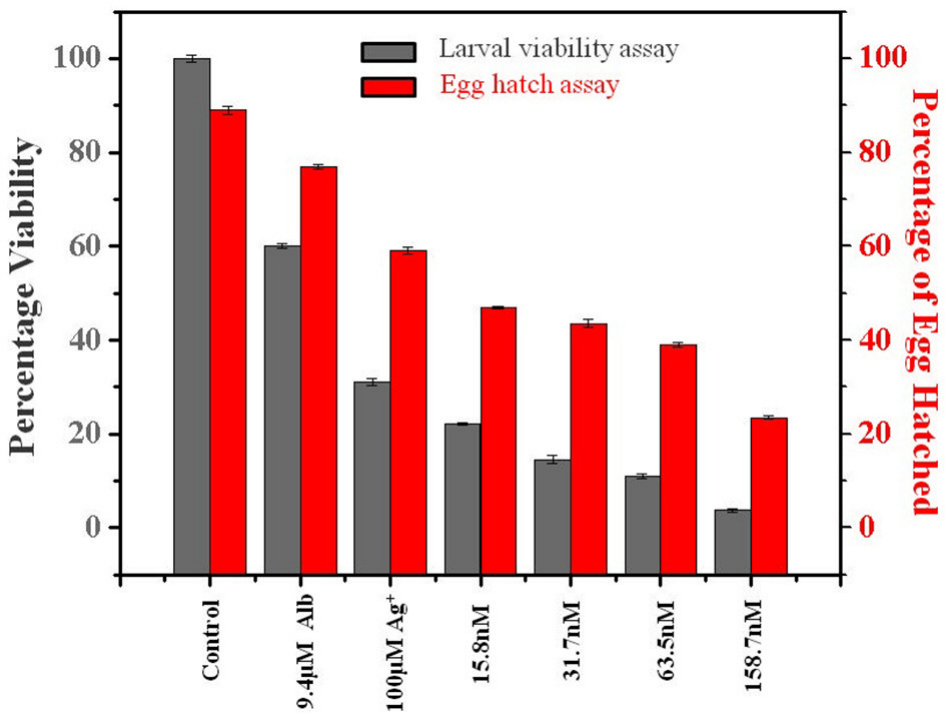
Percentage viability effect of *Lansium parasiticum*-protected silver nanoparticles (LAgNPs) on L3 larvae survival and hatching of *Haemonchus contortus* eggs. (Gray) Viability (survival) percentage of the larval stage L3 after 24 h of treatments with LAgNPs of different concentrations along with controls. (Red) Comparative graph showing the influence of LAgNPs on egg hatch. For 48 h, the treatment was performed using LAgNPs of different concentrations along with controls. In each well, around 200 eggs were taken, and the results of three independent experiments were presented as an average.

After 24 h of treatment with different concentrations of LAgNP and respective controls, movement of morphological changes like shrinkage and ruptured morphology was observed for just before L3 larvae. The concentration-dependent loss of viability of L3 larvae was prominent with LAgNPs. Survival of larvae at 33.3 ± 2.6, 29.5 ± 1.7, 22.2 ± 2.5, and 14.8 ± 2.1% was observed for 15.8, 31.7, 63.5, and 158.7 nM LAgNP treatment, respectively, whereas the recommended dose of Alb (9.4 μM) showed 60 ± 0.5% and Ag^+^ (100 μM) showed 31 ± 0.7% survival for 24 h of treatment (Figure 3). For larval mortality inhibition, LD_50_ has obtained 64.3 ± 8.5 nM for 24 h (Supplementary Figure 4C).

#### Egg Hatch Assay

After 48 h of treatment with LAgNPs and respective controls, the number of eggs hatched was calculated using Equation (2). The treatment concentration-dependent reduction of egg hatching efficiency was due to the LAgNP treatment; 32.1 ± 2.6, 45 ± 2.7, 47.2 ± 1.3, and 51.2 ± 1.9% reduction of egg hatching were observed in comparison with those of the control for 15.8, 31.5, 63.5, and 158.7 nM LAgNPs, respectively, whereas the recommended dose of Alb (9.4 μM) showed 13.5 ± 0.5% and Ag^+^ (100 μM) showed 33 ± 0.7% reduction of egg hatching or larvae (L1) formation (Figure 3). LD_50_ for inhibition of egg hatch was obtained at 144.4 ± 3.1 nM for 48 h (Supplementary Figure 4D).

### Ultra-Morphological Analysis for Tissue Damage Due to LAgNP Exposure

**Figure 4:**
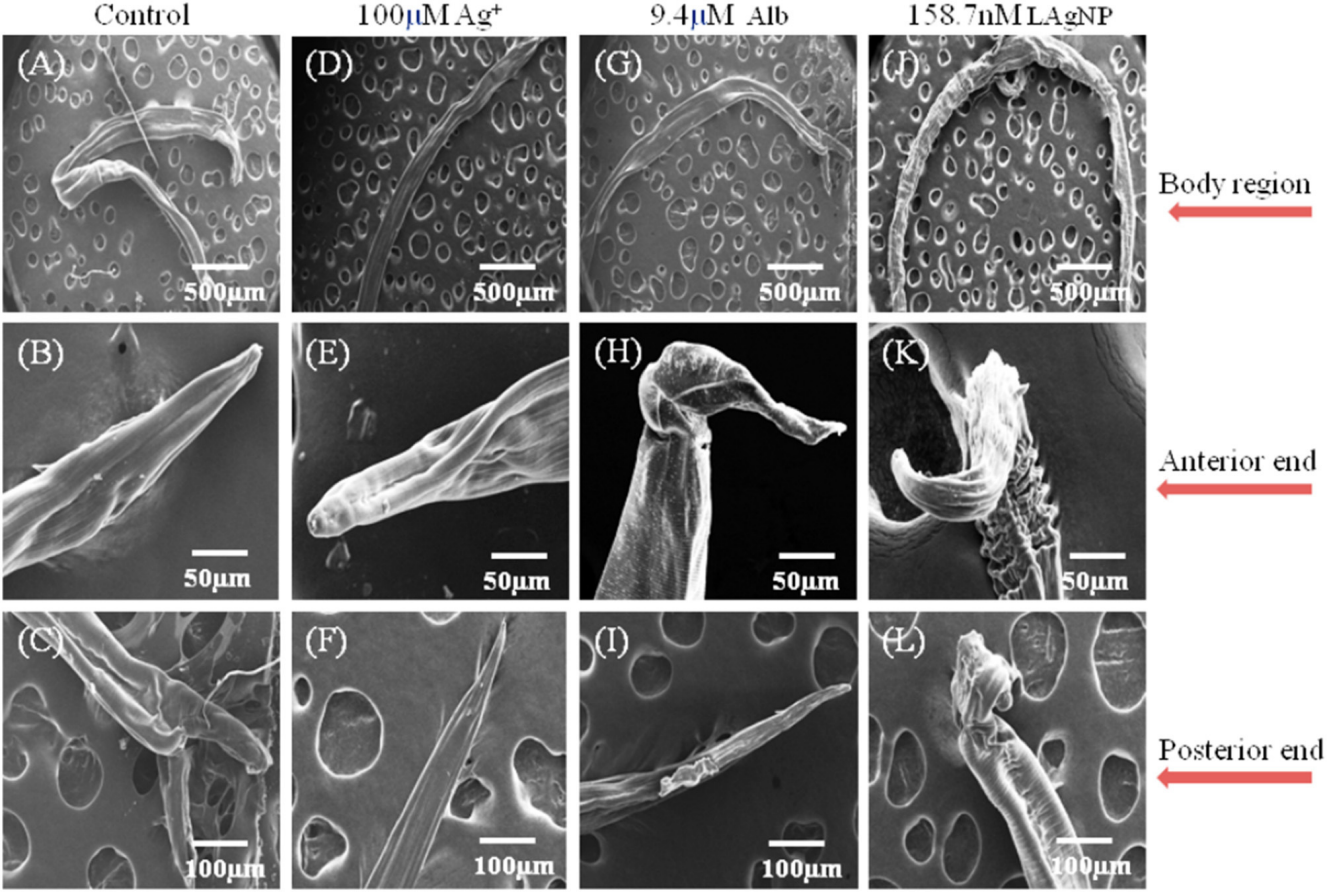
Study of ultramicroscopic morphological damage in adult *Haemonchus contortus* under the influence of *Lansium parasiticum*-protected silver nanoparticles (LAgNPs). The helminth was treated for 12 h, and after treatment, the helminths were dehydrated and gold-coated. Thereafter, the ultra-structures were observed using SEM at 15 (kV). **(A,E,I)** Treatment with control, RPMI media; **(B,F,J)** Ag^+^ (100 μM); and **(C,G,K)** 9.4 μM Alb-treated adult worms. **(D,H,I)** Adult worms after treatment with 158.7 nM LAgNPs. **(A–D)** Whole body of the control and Ag^+^-treated, Alb-treated, and LAgNP-treated worms, respectively. **(E–H)** Anterior end of the control and Ag^+^-treated, Alb-treated, and LAgNP-treated worms, respectively. **(I–L)** Posterior ends of the control and Ag^+^-treated, Alb-treated, and LAgNP-treated worms, respectively. LAgNP-treated worms showed profound damages to tissue architecture and loss of tissue integrity, with disorganization of the bursa regions.

The extent of physical damage of *H. contortus* was monitored using scanning electron microscopy. A complete comparative study for the changes in the morphology of tissue damage is shown in Figure 4, comparing control (RPMI Media), Alb (9.4 μM), Ag^+^ (100 μM), and 158.7 nM LAgNPs after 3 h of treatment. The complete body region of the adult worm is depicted in Figures 4A,D,G,J. The untreated *H. contortus* worm with a smooth cuticle and having a well-developed body region of an adult can be seen in Figures 4A,D,G, whereas in Figure 4J, a complete distortion of the outer morphology along with prominent shrinkage of the worm body can be observed. Figures 4B,E,H,K show the adult helminths' anterior region. Almost no distortions were observed in the control (Figure 4B). Partial and complete disruptions were observed in Ag^+−^, Alb–, and LAgNPs-treated (158.7 nM) worms, respectively (Figures 4C,H,K). Similar observations were prominent in the posterior ends of the control and treated worms as well (Figures 4C,F,I,L).

### Generation of Reactive Oxygen Species Stress Due to LAgNP Exposure

**Figure 5:**
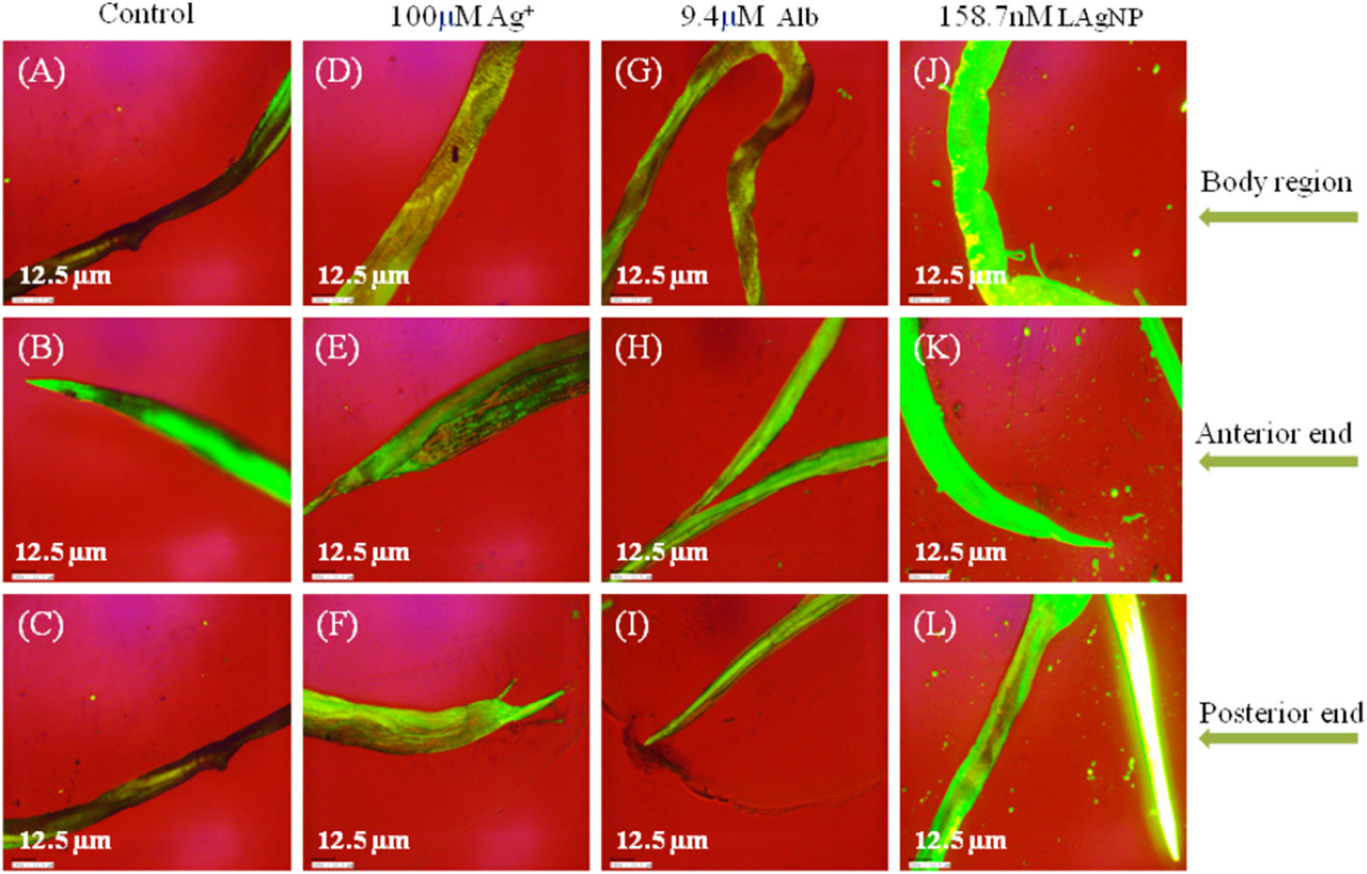
Generation of oxidative stress in adult worms due to exposure of *Lansium parasiticum*-protected silver nanoparticles (LAgNPs). Adult *Haemonchus contortus* were treated for 3 h with LAgNPs and controls and processed for reactive oxygen species (ROS) [staining with 2′,7′-dichlorofluorescein diacetate (DCFDA)]. **(A,E,I)** Amount of ROS in the body and in the anterior and posterior regions, respectively, of the adult helminth in the control, RPMI media. **(B,F,J)** Amount of ROS in the body and in the anterior and posterior regions, respectively, of the adult helminth in 100 μM Ag^+^. **(C,G,K)** Amount of ROS in the body and in the anterior and posterior regions, respectively, of the adult helminth when treated with 9.4 μM Alb. **(D,H,L)** Amount of ROS when treated with the highest concentration of LAgNPs (158.7 nM). After the desired treatment, the worms were incubated with 100 nM DCFDA in the dark for 20 min. Thereafter, the images were taken using an inverted fluorescence microscope by Dewinter, Italy.

Massive cellular stress response for ROS generation had been observed due to LAgNP (158.7 nM) treatment within 3 h. A complete comparative study for the changes in ROS generation of the stress response is shown in Figure 5, comparing control (RPMI media), Alb (9.4 μM), Ag^+^ (100 μM), and 158.7 nM LAgNPs after 3 h of treatment. In the control tissue (Figure 5), the low level of ROS generation showed a moderate level of ROS that was observed using DCFDA fluorescence. Figures 5A,D,G,J showed the complete body region of LAgNP-treated adult *H. contortus*, where A, D, and G showed a very less or partial generation of ROS, respectively, and showed a high generation of ROS. The generation of ROS was prominent in the anterior (Figures 5B,E,H) and the posterior ends (Figures 5C,F,I) when treated with control (RPMI media), Ag^+^, and Alb, but the high generation of oxygenated species was observed in K (anterior) and L (posterior) ends when treated with LAgNPs.

### Determination of Nitric Oxide Synthase

**Figure 6:**
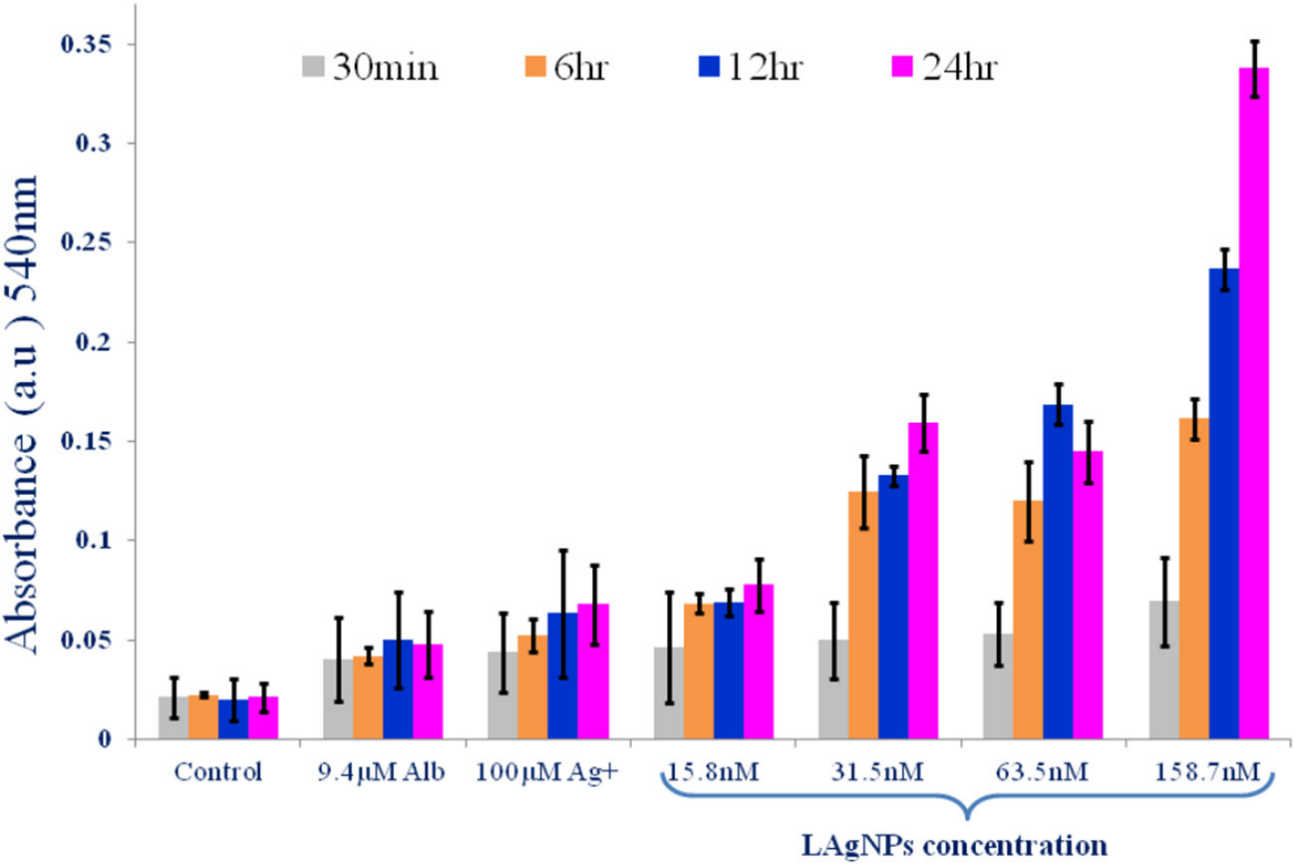
Alteration of activity of nitric oxide synthase (NOS) enzyme due to *Lansium parasiticum*-protected silver nanoparticle (LAgNPs) stress. A comparative graph showing the generation of nitric oxide free radicals as a result of NOS activity in *Haemonchus contortus* worms after treating with LAgNPs of different concentrations (158.7 nM) along with controls for 30 min and 6, 12, and 24 h.

The generation of NOS with the stress response in the worm due to LAgNP treatment was monitored using the Greiss reagent. The results showed a dose-dependent and subsequently time-dependent increase in the NOS level in the worms. The NOS level has increased from 0.0462 ± 0.028 to 0.081 ± 0.01 within 24 h for the lowest dose (15.8 nM) of LAgNPs, whereas the NOS response becomes further significantly prominent and increases from 0.07767 ± 0.01 and 0.351 ± 0.01 (within 24 h) due to 158.7 nM LAgNP treatment (Figure 6). The change of NOS level in response to Ag^+^ and Alb was moderate and not of high significance (Figure 6).

### Alteration of Catalase, Superoxide Dismutase, and Glutathione Peroxidase Activity in Response to LAgNP-Induced Oxidative Stress

The increase of reactive oxygen and nitrogen species due to LAgNP treatment generated huge oxidative stress within the worm's physiological system, which may alter the change in the activity of metabolic and stress-responsive enzymes. A steady enhancement in CAT activity with an increase of LAgNP concentration, resulting in an increase in stress response, had been observed. In different concentrations of LAgNP (15.8, 31.7, 63.5, and 158.7 nM), the amount of CAT enzyme was calculated as 4.75 ± 0.17, 5.35 ± 0.36, 7.9 ± 0.37, and 8.2 ± 0.4 U/mg proteins, respectively, after 3 h of treatment, in comparison with the 0.22 ± 0.05, 4.1 ± 0.37, and 0.52 ± 0.05 U/mg proteins for the untreated control, Alb (9.4 μM), and Ag^+^ (100 μM), respectively Table 3. A consistent SOD activity increment with an increase in stress response had also been observed. The SOD enzyme activity after the treatment was found to be 5.70 ± 0.4, 5.85 ± 0.17, 6.11 ± 0.26, and 6.60 ± 0.54 U/mg protein for LAgNPs 15.8, 31.7, 63.5, and 158.7 nM, respectively, in relation to the control where 4.8 ± 0.22 Alb and 0.56 ± 0.07 Ag^+^ were used. The untreated worms showed an SOD activity of 0.21 ± 0.018 U/mg of worm protein (Table 3). In contrast to other antioxidant activities, the activity of GPx also increases when the worms were exposed to LAgNPs' increasing concentrations. At 340 nm, spectrophotometrically, the amount of GPx was observed. The activity of the GPx enzyme was calculated using the Equation (3). The GPx enzymes' active amount was 2.5 ± 0.15, 2.6 ± 0.16, 2.73 ± 0.15, and 2.9 ± 0.16 U/mg protein for different concentrations of LAgNP (15.8, 31.7, 63.5, and 158.7 nM, respectively), in comparison with the control where the amount was 2.6 ± 0.25 U/mg proteins in Alb and 0.58 ± 0.058 U/mg proteins in Ag^+^. In the untreated worms, it was found to be 0.21 ± 0.071 U/mg proteins (Table 3).

**Table 3:** The toxicity of LAgNPs on *Haemonchus contortus* to hinder the oxidative stress response elements.

**Concentration of AgNPs**	**U/mg protein**			**μM/mg protein**
	**CAT**	**SOD**	**GPx**	**GSH**
Control	0.22 ± 0.05	0.21 ± 0.018	0.25 ± 0.07	0.28 ± 0.01
9.4 μM Alb	4.1 ± 0.37	4.8 ± 0.2	2.6 ± 0.25	1.4 ± 0.05
100 μM Ag^+^	0.56 ± 0.05	0.52 ± 0.07	0.58 ± 0.057	0.59 ± 0.009
15.8	7.9 ± 0.37	6.11 ± 0.26	2.73 ± 0.15	1.3 ± 0.14
31.7	8.2 ± 0.41	6.6 ± 0.54	2.9 ± 0.16	1.41 ± 0.10
63.5	8.36 ± 0.1	6.83 ± 0.20	3.2 ± 0.25	1.59 ± 0.11
158.7	10.7 ± 0.37	7.74 ± 0.89	3.3 ± 0.18	1.75 ± 0.05

*Helminths prevent the alteration of their metabolic functions due to the generation of oxide synthase-related stress. The toxicity of LAgNPs on helminths causes a reaction with a cellular molecule having metabolic functions that increase the regeneration of free radicals, which was induced due to the oxidative damage defense mechanisms to include enzymatic factors like (i) catalase (CAT), (ii) superoxide dismutase (SOD), (iii) glutathione peroxidase (GPx), and (iv) glutathione (GSH). The enzymatic activity of these factors was measured in terms of enzyme unit per milligram of protein (U/mg protein) for CAT, SOD, and GPx and (μM/mg protein) in GSH*.

### ROS Enhances Cellular Combat by Increasing the Concentration of Reduced Glutathione

In combat to ROS, cellular response induced by GSH causes oxidative stress in the worms when exposed to LAgNPs. A significant enhancement in the amount of GSH produced was measured in LAgNP-treated worms. An increase in GSH was observed (0.28 ± 0.05 μM/ mg for untreated) along with the increase of the dose of LAgNPs (15.8, 31.7, 63.5, and 158.7 nM) from 1.105 ± 0.025, 1.24 ± 0.06, 1.3 ± 0.141, and 1.41 ± 0.1078 μM/mg protein, respectively. The treatment of Alb also induced a significant increment in the GSH concentration (1.75 ± 0.050 μM/mg protein), and Ag^+^ causes 0.59 ± 0.009 μM/mg protein accumulation of GSH (Table 3).

## Discussion

Due to the shortage of drug availability and the development of resistance against existing drugs, there is an utmost necessity to develop new drugs for the treatment and the management of parasitic worm infections. This is the first report showing the anti-helminthic activity of *L. parasiticum* aqueous extract-protected silver nanoparticles. Here biocompatible, stable, and eco-friendly LAgNPs were synthesized using the green synthesis technique under the sunlight. *L. parasiticum* is a relatively less studied plant for medical purposes, although the use of seed extracts as anti-helminthic and anti-ulcer medication treatment had been reported in Vietnamese traditional medicine (Ruhisha and Choudhury, 2017; Potipiranun et al., 2018). Further use of bark extract had been reported for dysentery and malarial treatment (Ramadhan et al., 2018). Traditionally, the fruit extract of the same has also been reported for diarrhea treatment (Tanaka et al., 2002; Abdullah et al., 2005; Sunpapao et al., 2016). In spite of the abundance of production, the use of this plant for modern medication has not been explored to its true potential. *H. contortus* is resistant to albendazole, a widely used anti-helminthic drug of the benzimidazole group. Treatment of *H. contortus* with LAgNPs showed a rapid elevation of (within 3 h) ROS- and NOS-dependent stress in the worms' body. Due to the generation of stress, the worms undergo metabolic changes which were also evident by observing the change in the activity of ROS-neutralizing enzymes in the worm's body due to LAgNP treatment. As a control experiment, citrate-protected AgNPs showed limited anti-helminthic activity in comparison with LAgNPs, suggesting a significant role of the active principal component(s) of ALE. Although the active principal component(s) of ALE has not been identified yet, these findings open hope for the development of next-generation anti-helminthic drugs.

## Conclusion

This is the first report of the unprecedented anti-helminthic efficiency of *L. parasiticum* aqueous extract-protected AgNPs by increasing both ROS and NOS and thereby causing alteration of metabolic activity and physical damage in worm tissue. *H. contortus* shows high resistivity against albendazole, which was also consistent in our findings, whereas LAgNPs not only killed adult worms but also showed a significant effect of reducing larval viability and egg hatching. Henceforth, the findings are highly useful in the wake of the increasing problem of drug resistance to the commercially available anti-helminthic, increase of life quality, and reduction of healthcare cost, but further detailed studies are required to understand the active principal component(s), efficacy, and toxicity of the system in animal models. In addition to that, the utility of the formulations against other parasitic infections, including helminthic infections, needs to be evaluated in the future.

## Data Availability Statement

The original contributions presented in the study are included in the article/Supplementary Materials, further inquiries can be directed to the corresponding author/s.

## Author Contributions

VG, DC, and LS: conception and design of the study and revising the manuscript critically for important intellectual content. VG and PK: acquisition of data. VG, PK, DC, and LS: analysis and/or interpretation of data and approval of the version of the manuscript to be published. VG: drafting the manuscript. All authors certify that they have participated sufficiently in the work to take public responsibility for the content, including participation in the concept, design, analysis, writing, or revision of the manuscript.

## Conflict of Interest

The authors declare that the research was conducted in the absence of any commercial or financial relationships that could be construed as a potential conflict of interest.
