# Supplementary material for: Biomedical Evaluation of Lansium parasiticum Extract-Protected Silver Nanoparticles Against Haemonchus contortus, a Parasitic Worm
Source: Front Mol Biosci. 2020 Dec 17;7:595646. doi: 10.3389/fmolb.2020.595646 (PMC7773940; doi:10.3389/fmolb.2020.595646)
Supplement: Supplementary file 1 [file Data_Sheet_1.docx]

**Biomedical evaluation of *Lansium parasiticum* extract protected silver nanoparticles against *Haemonchus contortus*, a parasitic worm**

Vanshita Goel^1^, Pawandeep Kaur^1^, Lachhman Das Singla^2*^, Diptiman Choudhury^1*^

1; School of Chemistry and Biochemistry, Thapar Institute of Engineering and Technology, Patiala, Punjab- 147004, India.

2; Department of Veterinary Parasitology, Guru Angad Dev Veterinary and Animal Sciences University, Ludhiana, Punjab- 141001, India

Corresponding Email: [diptiman@thapar.edu](file:///C:\Users\hp\Downloads\diptiman@thapar.edu) and [ldsingla@gmail.com](file:///C:\Users\hp\Downloads\ldsingla@gmail.com)

**Short Title**: Antihelminthic activity of *L. parasiticum* nanoparticles.

**Supplementary Information:**

1. **Preliminary Phytochemical assay of *Lansium parasiticum***

Various plant extracts (hexane, chloroform, ethyl acetate, methanol, and aqueous) are tested for the presence of different phytochemicals. Following test are performed for the phytochemical essay.

**Test for Alkaloids**- The extract was dissolved in dilute HCl separately, filtered and filtrates were tested for the presence of alkaloids.

- Mayer’s test- The filtrates were treated with a few drops of Mayer’s reagent (2 gm I_2_ + 6 gm KI in 100 ml H_2_O). The formation of yellow-colored precipitates confirmed the presence of alkaloids.
- Wagner’s test- The filtrates were treated with a few drops of Wagner’s reagent (1.36 gm HgCl_2_ + 5 gm KI in 100 ml H_2_O). The formation of reddish/brown precipitates indicates the presence of alkaloids.

**Test for Carbohydrates**- The extract was dissolved in distilled water, filtered and filtrates were testes for the presence of carbohydrates.

- Benedict’s test- The filtrate was treated with a few drops of benedict’s reagent (10 gm Na_2_CO_3_ + 17.3 gm Na_3_C_6_H_5_O_7_+ 1.7 gm CuSO_4_). The formation of orange-red precipitates indicates the presence of carbohydrates.

**Test for Flavonoids**-

- Alkaline reagent test- Each extract was tested with 2 ml of NaOH. The formation of intense yellow color which disappears on the addition of an equal amount of acid indicates the presence of flavonoids.
- Lead acetate test- Each extract was treated with a few drops of lead acetate and the formation of a yellow precipitate indicates the presence of flavonoids.

**Test for Glycosides**-

- Modified Borntrager’s test- The extracts were treated with 1-2 ml of FeCl_3_ and then heated over the water bath for 5 minutes. This solution was allowed to cool and then extracted with equal volumes of benzene. The benzene layer was separated and treated with a few drops ammonia solution. Pink coloration indicates the presence of glycoside.

**Test for Saponins**-

- Froth test- The extracts were diluted with distilled water in a graduated cylinder up to 10 ml and this was shaken for 5 minutes. The formation of a foam layer of at least 1 cm indicates the presence of saponins.
- Foam test- 2-3 ml of extract was shaken with water in a test tube for 5 minutes. If the foam persisted for 10 minutes, it indicates the presence of saponins.

**Test for Phenols**-

- Ferric Chloride test- The extracts were treated with 1-2 ml of FeCl_3_ solution. The formation of bluish-black precipitates indicates the presence of phenols.

**Test for Tannins**- The extracts were dissolved in 5 ml distilled water for the following tests and filtered.

- Lead Acetate test- To 1-2 ml of filtrate a few drops of 1% lead acetate were added. The formation of yellow precipitates infers the presence of tannins.
- FeCl_3_ test- To 1-2 ml filtrates, a few drops of 5% FeCl_3_ was added. The formation of a green precipitate indicates the presence of tannins.

**Test for Steroids**- Each extract was dissolved in 5 ml chloroform and filtered. The filtrate was analyzed for the presence of steroids.

- Salkowaski’s Test- The filtrate was treated with a few drops of sulphuric acid, shaken for a few minutes, and allowed to stand undisturbed for some time. The appearance of a golden yellow coloration indicates the presence of steroids.
- LibermannBurchard’s test- The filtrate was treated with a few drops of acetic anhydride. The solution was boiled and then allowed to cool. To this a few drops of Conc. Sulphuric acid was added. The appearance of a brown ring at the junction indicates the presence of steroids.

**Test for Proteins and Amino Acids-**

- Xanthoprotic test- The extracts were treated with a few drops of nitric acid. The appearance of a yellow color indicates the presence of proteins.

**Test for Oils and Fats-**

- Filter paper test- Different extracts were pressed between filter papers. An oil stain on the filter paper indicates the presence of oils and fats.

**Results:**

**Table S1.** Phytochemical Analysis

| **S. No.** | **Test Performed** | **Hexane Extract** | **Chloroform Extract** | **Petroleum**  **Ether** | **Ethyl Acetate Extract** | **Methanol Extract** | **Aqueous Extract** |
| --- | --- | --- | --- | --- | --- | --- | --- |
| **1** | **Alkaloids** |  |  | | | | |
| Mayer’s Test  Wagner’s Test | | - | + | + | + | + | + |
|  |  | - | + | + | + | + | + |
| **2.** | **Carbohydrates** |  |  | | | | |
| Benedict’s Test | | - | + | - | - | + | + |
| **3.** | **Flavanoids** |  |  | | | | |
| Alkaline R. Test  Lead Acetate Test | | - | + | + | - | + | - |
|  |  | - | + | + | - | + | - |
| **4.** | **Glycosides** |  |  | | | | |
| Borntrager’s Test | | - | - | - | - | - | - |
| **5.** | **Saponins** |  |  | | | | |
| Froth Test  Foam Test | | - | - | - | - | + | + |
|  |  | - | - | - | - | + | + |
| **6.** | **Phenols** |  |  | | | | |
| FeCl_3_ Test | | - | - | - | + | + | + |
| **7.** | **Tannins** |  |  | | | | |
| Lead Ac. Test  FeCl_3_ Test | | - | - | - | - | + | + |
|  |  | - | - | - | - | + | + |
| **8.** | **Steroids** |  |  | | | | |
| Salkowaski Test  Libermann’s Test | | + | + | - | + | - | + |
|  |  | + | + | - | + | - | + |
| **9.** | **Proteins** |  |  | | | | |
| Xanthoprotic Test | | - | - | - | - | - | + |
| **10.** | **Oils and Fats** |  |  | | | | |
| Filter Paper Test | | - | - | - | - | + | + |

**Table S2.** Percentage composition of Phytochemical Analysis.

| **Phytochemicals** | **% composition (w/w)** |
| --- | --- |
| Alkaloids | 0.64 |
| Flavonoids | 2.56 |
| Terpenoids | 0.52 |
| Tannins | 1.55 |

**Table S3.** The stability and metal leaching of LAgNPs Inductively coupled plasma atomic emission spectroscopy (ICP-AES), (AGILENT, 4100) studies were performed after the aging of samples up to 6 months. The maximum amount of around ~30% Ag^+^ release was observed after 6 months of aging. LAgNPs lasted for 6 months and found a maximum of ~ 30% release of silver ions in the solution. Therefore 30% silver solution was kept as the control for most of the experimental sets.

| **Parameters** | **Results for Ag^+^ leaching for LAgNPs** | | | |
| --- | --- | --- | --- | --- |
| Time of Aging (months) | **0** | **2** | **4** | **6** |
| Silver ions (Ag^+^) in mg/l | 0.1 ± 0.1 | 1.27 ± 0.567  (~5 %) | 3.24 ± 0.891  (~12 %) | 7.72 ± 1.458  (~30 %) |

**Table S4.** Table showing the p-values for the Egg Hatch Assay and Larval Motility Assay (L-3) of *H. contortus* worms when treated with different concentrations of LAgNPs (15.8nM, 31.7nM, 63.5nM and 158.7nM). Statistical analysis of data was conducted by a Student’s t-test, by using MS Excel, and two measurements were statistically significant if the corresponding p-value was <0.01.

|  | **15.8nM** | **31.7nM** | **63.5nM** | **158.7nM** |
| --- | --- | --- | --- | --- |
| **Egg Hatch Assay** | 1.38355E-11 | 2.18651E-11 | 1.13407E-10 | 6.27186E-10 |
| **Larval Motility Assay** | 5.50957E-13 | 2.37401E-12 | 9.97562E-12 | 7.93434E-11 |

**Table S5.** Table showing the p-values for the enzymatic activity of different catalysts SOD, CAT, GPx, GSH of adult *H. contortus* worms when treated with different concentrations of LAgNPs (15.8nM, 31.7nM, 63.5nM and 158.7nM**)**. Statistical analysis of data was conducted by a Student’s t-test, by using MS Excel, and two measurements were statistically significant if the corresponding p-value was <0.01.

| **Enzymatic Activity** | **Superoxide Dismutase (SOD)** | **Catalase (CAT)** | **Glutathione Peroxidase (GPx)** | **Glutathione (GSH)** |
| --- | --- | --- | --- | --- |
| **15.8nM** | 3.62694E-13 | 2.74722E-11 | 5.2248E-13 | 1.62E-11 |
| **31.7nM** | 2.5069E-12 | 4.51635E-11 | 2.57836E-12 | 3.17E-11 |
| **63.5nM** | 1.65888E-11 | 5.05788E-11 | 1.22419E-11 | 8.49E-11 |
| **158.7nM** | 1.41278E-10 | 4.43158E-10 | 8.71562E-10 | 3.84E-10 |

**Table S6.** Table showing the p-values for nitric oxide synthase (NOS) activity of *H. contortus* worms when treated with different concentrations of LAgNPs (15.8nM, 31.7nM, 63.5nM, and 158.7nM**)**. Statistical analysis of data was conducted by a Student’s t-test, by using MS Excel, and two measurements were statistically significant if the corresponding p-value was <0.01.

|  | **15.8nM** | **31.7nM** | **63.5nM** | **158.7nM** |
| --- | --- | --- | --- | --- |
| **6hr** | 8.21842E-10 | 6.22619E-10 | 4.13012E-10 | 1.76271E-10 |
| **12hr** | 9.49E-10 | 8.7E-10 | 8.13E-10 | 4.35E-10 |
| **24hr** | 1.74E-09 | 1.64E-09 | 1.7E-09 | 1.62E-09 |

**

**Fig S1:** Color changes under the sunlight during formation of LAgNPs using aqueous *L. parasiticum* extract (ALE) as a reducing agent. The pictures were taken at different time intervals (A) 0 min (B) 5 min (C) 10min (D) 20min and (E) 30min of sunlight exposure.

**Fig S2. Physical characterization of citrate protected AgNPs:** (A) Surface plasmon resonance spectra of citrate reduced AgNPs. The spectra were scanned in the range of 300 nm-700 nm. (B) Dynamic light scattering study showing the distribution of hydrodynamic diameter and disparity of the citrate coated AgNPs. The hydrodynamic size obtained was 23 nm.

Toxicity of LAgNPs on the human normal kidney cells and *H. contortus* cells:

The cell viability results showed mild toxicity of LAgNPs on HEK293 cells for 24 hrs exposure. At the lowest concentration (15.8 nM), there were no significant changes were observed in HEK293 viability. With an increase in LAgNPs concentration, however, the percentage of viable cells was observed (77.5 ± 5.06 %, 71.3 ± 9.8 % and 62.1 ± 9.3 %) for 31.7, 63.5 and 158.7nM treatments respectively. Further, silver nanoparticles are known for their leaching effect, and a maximum of ~30% (70 μM) silver leaching was observed upon 6 months storage of silver nanoparticles (**ST3**). Therefore, we have also checked toxicity caused by the leaching effect HEK293 cells were treated with various concentrations (50-250 μM) of Ag^+^ for 24 hrs. 76.5 ± 8.8%, 72.5 ± 5.8%, and 68.4 ± 0.2% cells were found to be alive due to (50, 100 and 250 μM) of Ag^+^ treatment for 24 hrs (**Fig. S3A**). On the contrary, *H. contortus* cells showed high susceptibility in the presence of LAgNPs. A sharp loss of cell viability (28.2± 7.3, 32± 12.4, 58.6 ± 3.3, and 68.8 ± 16.6 % loss of cell viability was observed for (15.8, 31.7, 63.5, 158.7 nM LAgNPs treatment respectively (**Fig. S3B**).

**Fig. S3 Cell viability study of humans and adult *H. contortus* upon exposure to LAgNPs**. (A) Human embryonic kidney (HEK239) cells were treated with various concentrations of LAgNPs and different concentrations of Ag^+^ (100µM) for 24 h and thereafter MTT assay was performed to determine the viability of cells. LAgNPs showed mild toxicity on HEK239 cells upon 24 h exposure. With a maximum dose of 158.7nM, 62.1 ± 9.3% cells died after 24 h in LAgNPs, whereas 60.5 ± 2.9% cells died because of 24 h treatment of 100µM Ag^+^. (B) ***H. contortus* were treated with various concentrations of LAgNPs** (15.8, 31.7, 63.5, and 158.7nM) for 3 hrs, and thereafter MTT assay was performed to determine the viability of cells. LAgNPs showed higher toxicity on worm cells upon 3 hrs exposure. Upon exposure to LAgNPs just for 3 hrs worms showed 72 ± 8.3 %, 68 ± 15.4 %, 41.6 ± 3.3 and 31.2 ± 16.6 % respectively.

The half minimal inhibitory concentration (LD_50_) was measured against the death percentage of adult *H. contortus*, (L- 3) larvae and egg hatch assay.

**Fig S4.** LD_50_ values of LAgNPs against various forms of *H. contortus*: In (A) Male death percentage in 12 hrs (B) Female death percentage (C) Larval Motility assay in 24 hrs and (D) Egg hatch assay in 48 hrs.
